# Complete mitochondrial genome of the Rufous burrowing snake, *Achalinus rufescens* (Reptilia: Xenodermatidae)

**DOI:** 10.1080/23802359.2017.1347842

**Published:** 2017-07-18

**Authors:** Yong Zhang, Dian-Cheng Yang, Li-Fang Peng, Aijun Jin, Shuangquan Duan, Song Huang

**Affiliations:** a School of Sciences, Tibet University, Lhasa, China;; b College of Life and Environment Sciences, Huangshan University, Huangshan, China;; c Administrative Office of Huangshan Nature Reserve, Huangshan Forestry Bureau, Huangshan, China

**Keywords:** Mitochondrial genome, Xenodermatidae, *Achalinus rufescens*

## Abstract

The complete mitochondrial genome (mitogenome) sequence of *Achalinus rufescens* was determined by using a PCR-based method. The total length of mitogenome is 17,339 bp and contains 13 protein-coding genes, 22 tRNA genes, 2 ribosome RNA genes and 2 control regions (D-loop). All the protein-coding genes of *A. rufescens* were distributed on the H-strand, except for the ND6 subunit gene and eight tRNA genes which were encoded on the L-strand. The phylogenetic tree of *A. rufescens* and 11 other closely species was built. The DNA data presented here will be useful to study the evolutionary relationships and genetic diversity of *A. rufescens*.

The Rufous burrowing snake (*Achalinus rufescens*) belongs to the family Xenodermatidae (Vidal et al. 2007; Zaher et al. 2009; Pyron et al. 2013; Cai et al. 2015), which distributed in China and Northern Vietnam (Orlov et al. 2000; Zhao 2006). There are nine species in the genus *Achalinus* presently. A few molecular data of this genus were determined. The complete mitochondrial genomes of *Achalinus meiguensis* have been reported (Wang et al. 2009). In this paper, we determined and described the complete mitogenome of *A. rufescens* in order to obtain basic genetic information about this species, enrich the *Achalinus* species genome resource and promote the further research concerning related species.

The specimen of *A. rufescens* (Voucher number: HS14023) was collected from Qimen County, Anhui Province, China (29°49′08″N, 117°32′17″E) as a new record of Anhui Province (Peng and Huang 2015). The complete mitogenome of *A. rufescens* (GenBank accession number KT897595) was sequenced to be 17,339 bp which consisted of 13 typical vertebrate protein-coding genes, 22 transfer RNA (tRNA) genes, 2 ribosomal RNA (rRNA) genes and 2 D-loops, which is similar to the typical mtDNA of snakes and other vertebrates (Boore 1999; Sorenson et al. 1999).

Most of the *A. rufescens* mitochondrial genes are encoded on the H-strand except for the ND6 gene and eight tRNA genes, which are encoded on the L-strand. The positions of RNA genes were predicted by the MITOS (Bernt et al. 2012), and the locations of protein-coding genes were identified by comparing with the homologous genes of other closely related species. The overall base composition of the entire genome was as follows: A (31.1%), T (26.4%), C (29.4%) and G (13.3%), which the percentage of A + T (57.5%) reflected a typical sequence feature of the vertebrate mitogenome. Among the mitochondrial protein-coding genes, the ATP8 was the shortest, while the ND5 was the longest. Eight of the 13 protein-coding genes (ND2, COII, ATPase 6, COIII, ND4L, ND4, ND5 and CYT b) initiate with ATG as start codon, while COI and ATPase 8 genes initiate with GTG, ND3 gene begin with ATT, and the other two protein-coding genes used ATA as start codon. Seven genes (COI, ATPase 8, ATPase 6, ND4L, ND4, ND5 and ND6) end with complete stop codons (AGG, TAA and AGA), and the other six genes end with T as the incomplete stop codons, which were presumably completed as TAA by post-transcriptional polyadenylation (Anderson et al. 1981). The 22 tRNA genes range in size from 52 to 73 bp. The 12S rRNA (926 bp) and 16S rRNA (1 489 bp) are located between the tRNA-Phe and ND1 genes and separated by the tRNA-Val gene. The D-loop1 of the *A. rufescens* mitogenome in size is 1129 bp and was located between the tRNA-Ile and tRNA-Leu genes, and the D-loop2 is 1122 bp long, between the tRNA-Pro and tRNA-Phe genes.

In order to validate the new determined sequences, we selected the 12 protein-coding genes located on heavy strand except for ND6 which encoded on the light strand of *A. rufescens* in this study and together with other 11 closely related species from GenBank to perform phylogenetic analysis. These species were as follows: *A*. *meiguensis*, *Cylindrophis ruffus*, *Deinagkistrodon acutus*, *Crotalus horridus*, *Ovophis okinavensis*, *Protobothrops dabieshanensis*, *P. jerdonii*, *Python regius*, *Viridovipera stejnegeri*, *Cryptelytrops albolabris* and *Charina trivirgata*. A maximum likelihood (ML) tree was constructed based on the dataset by online tool RAxML (Stamatakis et al. 2008) (Figure 1). The phylogenetic analysis result was consistent with the previous research. It indicated that our new determined mitogenome sequences could meet the demands and explain some evolution issues.

**Figure 1.**
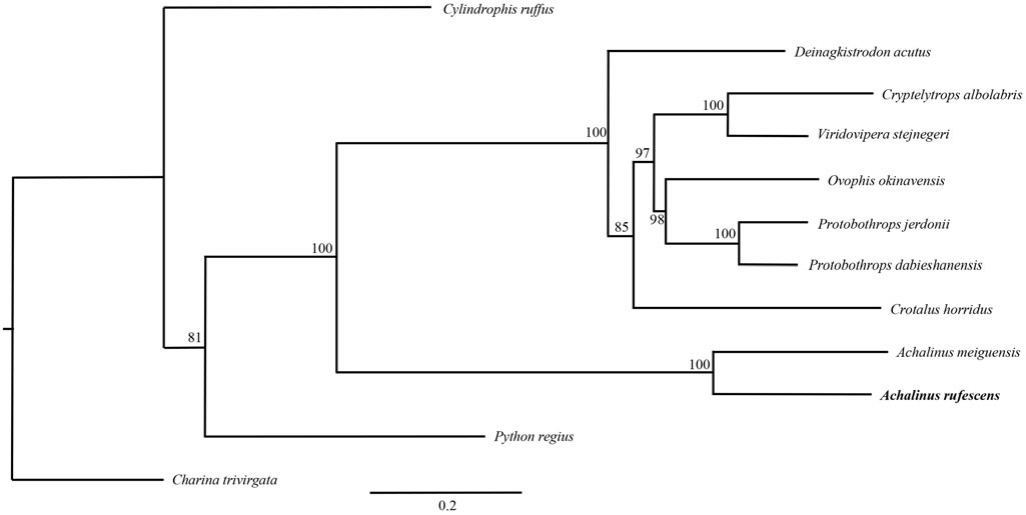
A maximum likelihood (ML) tree of *Achalinus rufescens* in this study and other 11 closely related species was constructed based on the dataset of 12 concatenated mitochondrial protein-coding genes by online tool RAxML. The numbers above the branch meant bootstrap value. Bold black branches highlighted the study species and corresponding phylogenetic classification. The analyzed species and corresponding NCBI accession numbers were as follows: *Cylindrophis ruffus* (AB179619), *Deinagkistrodon acutus* (EU913476), *Cryptelytrops albolabris* (KF311102), *Viridovipera stejnegeri* (FJ752492), *Ovophis okinavensis* (AB175670), *Protobothrops jerdonii* (KF003004), *P. dabieshanensis* (KC112560), *Crotalus horridus* (HM641837), *Achalinus meiguensis* (KT897594), *A. rufescens* (KT897595), *Python regius* (AB177878) and *Charina trivirgata* (GQ200595).
